# Formation of intracellular vesicles within the Gram^+^ *Lactococcus lactis* induced by the overexpression of Caveolin-1β

**DOI:** 10.1186/s12934-022-01944-9

**Published:** 2022-11-17

**Authors:** A. Flourieusse, P. Bourgeois, E. Schenckbecher, J. Palvair, D. Legrand, C. Labbé, T. Bescond, L. Avoscan, S. Orlowski, A. Rouleau, A. Frelet-Barrand

**Affiliations:** 1 FEMTO-ST Institute, UMR 6174, CNRS, Bourgogne Franche-Comté University, 15B Avenue Des Montboucons, 25030 Besançon Cedex, France; 2 Agroecology Agro Dijon Institute, CNRS, INRAE, DImaCell, Burgundy University, Bourgogne Franche-Comté University, Dijon, France; 3 CEA/Institut Joliot/SB2SM, CNRS/I2BC (UMR9198), Université Paris-Saclay, Gif-Sur-Yvette, France

**Keywords:** *Lactococcus lactis*, Membrane proteins, Caveolin-1, Intracellular vesicles

## Abstract

**Background:**

Caveolae are invaginated plasma membrane domains of 50–100 nm in diameter involved in many important physiological functions in eukaryotic cells. They are composed of different proteins, including the membrane-embedded caveolins and the peripheric cavins. Caveolin-1 has already been expressed in various expression systems (*E. coli,* insect cells, *Toxoplasma gondii,* cell-free system), generating intracellular caveolin-enriched vesicles in *E. coli*, insect cells and *T. gondii*. These systems helped to understand the protein insertion within the membrane and its oligomerization. There is still need for fundamental insights into the formation of specific domains on membrane, the deformation of a biological membrane driven by caveolin-1, the organization of a caveolar coat, and the requirement of specific lipids and proteins during the process. The aim of this study was to test whether the heterologously expressed caveolin-1β was able to induce the formation of intracellular vesicles within a Gram^+^ bacterium, *Lactococcus lactis*, since it displays a specific lipid composition different from *E. coli* and appears to emerge as a good alternative to *E. coli* for efficient overexpression of various membrane proteins.

**Results:**

Recombinant bacteria transformed with the plasmid pNZ-HTC coding for the canine isoform of caveolin-1β were shown to produce caveolin-1β, in its functional oligomeric form, at a high expression level unexpected for an eukaryotic membrane protein. Electron microscopy revealed several intracellular vesicles from 30 to 60 nm, a size comparable to *E. coli* h-caveolae, beneath the plasma membrane of the overexpressing bacteria, showing that caveolin-1β is sufficient to induce membrane vesiculation. Immunolabelling studies showed antibodies on such neo-formed intracellular vesicles, but none on plasma membrane. Density gradient fractionation allowed the correlation between detection of oligomers on Western blot and appearance of vesicles measurable by DLS, showing the requirement of caveolin-1β oligomerization for vesicle formation.

**Conclusions:**

*Lactococcus lactis* cells can heterologously overexpress caveolin-1β, generating caveolin-1β enriched intracellular neo-formed vesicles. These vesicles might be useful for potential co-expression of membrane proteins of pharmaceutical interest for their simplified functional characterization.

**Supplementary Information:**

The online version contains supplementary material available at 10.1186/s12934-022-01944-9.

## Introduction

Caveolae are flask-shaped invagination of the plasma membrane of mammalian cells of 50 to 100 nm of diameter [1]. Involved in many physiological functions (mechanosensing, signalling, endocytosis, lipid homeostasis), they are highly abundant in muscle cells, adipocytes, endothelial cells and fibroblasts, and linked to many diseases including cardiovascular diseases, cancers, degenerative muscular dystrophies and kidney diseases [2–6]. Caveolae are essentially linked to the presence in the plasma membrane of the caveolin proteins, mainly caveolin-1 and -3 [7], and cavins (cavin1-4) [8]. Deletion of either caveolin-1 or cavin-1 leads to the loss of caveolae. Both N- and C-termini of caveolin-1 (21–24 kDa) have been shown to reside within the cytoplasm, the N-terminus segment harboring the oligomerization domain [9] while the intramembrane domain, with its helix-break-helix motif, is believed to contribute to the formation of a curved membrane thanks to a wedge effect [10]. The protein undergoes multiple modifications during membrane trafficking from synthesis site to plasma membrane [8], including oligomerization which appeared shortly after biosynthesis and association with lipids [11, 12]. Caveolin oligomerization and formation of membrane domains enriched in cholesterol, phosphatidylserine and glycosphingolipids, in the presence of cavins, are believed to be critical for caveolae formation in mammalian cells [1, 13–15].

Caveolin-1, isoforms αand β, have already been expressed in various heterologous expression systems. These systems helped to provide relative high amounts of protein, which are necessary to perform structural and functional studies, to understand the way the protein is inserted within the membrane and how it undergoes oligomerization. Moreover, it is also essential to provide fundamental insights into the formation of specific domains on membrane, the deformation of a biological membrane driven by caveolin-1, the organization of a caveolar coat, and the requirement of specific lipids and proteins during the process. Caveolin-1, isoforms αand β have been expressed in *E. coli* [16, 17]*,* insect cells [18–20], *Toxoplasma gondii* [21], and cell-free system [22]. In all these systems (except cell-free), the expression of the recombinant caveolin induced the formation of intracellular vesicles within the heterologous host. Notably, in these few expression systems, both whole and truncated versions of caveolin-1 have been demonstrated to be sufficient alone to promote membrane budding and protrusion, and the eventual formation of intracellular vesicles presenting high homogeneity of size and shape, provided local concentration of the protein is high enough [23]. Such intracellular vesicles, called “heterologous caveolae” [17], could be used for diverse biotechnological purposes, and especially for co-expression of other membrane proteins (MPs) of interest [20, 24].

The various expression systems available for heterologous expression of MPs are either prokaryotic, such as the most common *E. coli*, or eukaryotic (yeasts, insect cells, mammalian cells). They display different features in terms of lipid composition and cellular machinery, with respective advantages and drawbacks while expressing MPs. Among them, the bacterial expression system *Lactococcus lactis* has emerged since 2000 as a good alternative to *E. coli* for expression of MPs, in particular for eukaryotic MPs [25–29]. Indeed, in contrast to *E. coli*, it displays interesting features for expression and further studies of MPs: it does not form inclusion bodies [26] and has only one membrane, that presents a specific lipid composition. Moreover, a tightly controlled system (NICE, NIsin-Controlled gene Expression [30]), based on the use of sub-inhibitory amounts of the antimicrobial compound nisin, has already been successfully used for functional expression and characterization of MPs from diverse origins (plants, bacteria, and mammals) and functional families (ABC transporters, mitochondrial carriers and others; for review, see [29]).

Here, we describe the heterologous expression of caveolin-1β in *L. lactis*, using the NICE system, and analyze whether the protein was able to induce the formation of intracellular vesicles within this Gram^+^ bacterium.

## Material and methods

### Bacterial strains and growth conditions

The strains used in this study are listed in Table 1. Lactococcal strains were grown on M17 medium (BK012HA, Biokar Diagnostics) supplemented either with 0.5% glucose (M17G medium) at 30 °C without shaking for DNA isolation or with 1% glucose (M17G1) at 30 °C with gentle shaking (90 rpm) for induction of expression. *E. coli* strains were grown in Luria–Bertani (LB) medium (L3522, Merck) at 37 °C with shaking (180 rpm). Antibiotics were used for plasmid maintenance at the following final concentrations: chloramphenicol (10 µg/mL) for *L. lactis* and kanamycine (100 µg/mL) for *E. coli*.

**Table 1:** Bacterial strains and plasmids used in this study

		Relevant genotype or phenotype	References or sources
Strains			
*E. coli*			
	DH5α	F- φ80lacZΔM15 Δ(lacZYA-argF)U169 recA1 endA1 hsdR17(rk-, mk+)	Invitrogen
		phoA supE44 thi-1 gyrA96 relA1 λ-	
*L. lactis*			
	NZ9000	MG1363 pepN::nisRK	Ozyme
	NZ9700	Progeny of the conjugation between nisin producer strain NIZO	NIZO
		B8 with MG1614 (RifR StrpR derivative of MG1363). Nisin producer	
		strain for induction experiments	
Plasmids			
pKL-HTC	Plasmid containing the caveolin gene	Kanr	Collaborators
pNZ8148		Chlr	Ozyme

Kanr and Chlr: resistance to kanamycine and chloramphenicol, respectively

### Cloning for caveolin-1β expression in pNZ8148

The gene of canine caveolin-1β (Cav1β, Uniprot P33724.2, 32–178) cloned into the pKL vector [20] with a 10-His affinity tag and TEV protease site at the N-terminus of the gene (HTC for His-tag-TEV-caveolin) was used for subcloning into pNZ8148. First, a mutation was performed to insert the restriction site *Nco*I at the start codon using the QuickChange Lightning Site-Directed Mutagenesis kit (Agilent) following the manufacturer’s instructions with the primer pKL-NcoIm fwd and rev (Table 2). The kanamycin-resistant clones obtained were tested by digestion with *Nco*I and *Nde*I (NEB, Ipswich, USA) after extraction using the Nucleospin Plasmid kit (Macherey–Nagel) and following the manufacturer’s instructions. The corresponding cDNA was excised from mutated pKL/HTC by digestion with *Nco*I and *Sac*I (NEB, Ipswich, USA) following the manufacturer’s instructions and ligated into pNZ8148NS previously digested with the same endonucleases. The two ligation reactions were purified, eluted, and then used to transform NZ9000 strain by electroporation [31]. Chloramphenicol-resistant clones were selected on M17GChl Agar Petri dishes after 1–2 days at 30 °C. Presence of the cDNA and correct sequence of the clones were confirmed by both endonuclease digestion and sequencing analysis with pNZ8148 fwd and rev primers (Table 2). The recombinant vector was termed pNZ-HTC.

**Table 2:** Oligonucleotides primers used for cloning and sequencing

Oligonucleotides	Sequence 5′-3′
pKL-NcoIm fwd	GGGCGCGGATCC**ATG***GGA*CATCATCATCATCATC
pKL-NcoIm rev	GATGATGATGATGATG*TCC***CAT**GGATCCGCGCCC
pNZ8148 fwd	CGCGAGCATAATAAACGGCTCTG
pNZ8148 rev	GTGTTGCTTTGATTGATAGCCAAAAAGC

### Induction of membrane protein expression

Precultures in M17G1Chl inoculated with frozen stock from both C- (negative control corresponding to bacteria transformed with the empty vector) and HTC recombinant bacteria were incubated overnight at 30 °C with gentle shaking (90 rpm). They were added at 1/40 (v:v) to 250 mL M17G1Chl in Schott bottles and incubated at 30 °C with gentle shaking (90 rpm) until the OD reached 0.75–0.80 (for details, see [31]). At this time, induction was performed by addition of nisin obtained from supernatant of culture of NZ9700 strains [32]. Incubation were pursued for 4 additional hours, optimal duration of induction suitable for higher level of MP expression in *L. lactis* [27–33]. Bacteria were harvested by centrifugation at 5000 g for 15 min at 4 °C. The pellets were resuspended, washed in buffer TN (50 mM Tris–HCl, 150 mM NaCl, pH 7.5) and centrifuged at 5000 g for 15 min, at 4 °C one more time. The bacterial pellets were kept at − 80 °C after resuspension in TN until isolation.

### Isolation of crude bacterial membrane proteins

The bacteria were disrupted by twofold passages through a One Shot (Constant Cell Disruption Systems, Northants, UK) at 35,000 p.s.i. (2.3 kbars) and kept on ice until centrifugation. After cell breakage, the lysates were centrifuged at 10,000 *g* for 10 min, at 4 °C, and the supernatant containing proteins was transferred into centrifuge tubes for further ultracentrifugation at 150,000 *g* for 1 h, at 4 °C. MPs present in pellets were resuspended in TN/1% glycerol and kept at − 80 °C.

### SDS–polyacrylamide gel electrophoresis and western blotting

Protein content of membrane fractions were estimated using the Bio-Rad protein assay reagent (Bio-Rad, Hercules, CA). Equal quantity of protein of each sample was mixed with 4 × sample buffer and heated at 95 °C for 10 min before separation by SDS polyacrylamide gel electrophoresis using a 4–12% gradient gel at the same time as a positive control (15 µg) corresponding to total MPs isolated from Sf21 insect cells transformed with the pKL/HTC plasmid [20] and expressing caveolin-1β at about 3% of total MPs (personal communication). For Western blotting, proteins were transferred to nitrocellulose membranes (10,600,019, Amersham), and blocked using 5% skim milk. Membranes were incubated either with antibody against caveolin (1/7000; 610,407, BD Science, USA) and then with secondary anti-mouse HRP-conjugated antibodies (1/3000; 170–6516, Bio-Rad, USA) or with His-HRP conjugate (1/5000; 15,165, Fisher). Detection was then performed through enhanced chemiluminescence (ECL) with a ChemiDoc system (Bio-Rad, USA). Bands corresponding to the proteins were analyzed and quantified through ImageLab software.

### Density gradient ultracentrifugation

First, 1.25 mg of total membrane proteins isolated from both C- and HTC recombinant bacteria cultured and isolated at the same time were loaded on a discontinuous sucrose gradient from 20 to 44%. After ultracentrifugation at 150,000 × *g* for 19 h at 4 °C, 24 fractions of 500 µL were then collected. Each fraction was divided into aliquots before storage at − 80 °C before further analysis. Sucrose density was measured for each fraction using a refractometer (Carl Zeiss, 47,729), and appeared to be almost similar for both C- and HTC fractions from 1 to 24; linear regression gave equivalent coefficients and R^2^ superior to 0.98.

### Dynamic light scattering (DLS) analysis

Particle diameters were measured by DLS (NanoZS, Malvern) with a 633 nm laser. Measurements were taken on samples diluted 1:100 (v:v) in PBS1x using cuvette (ZEN0040, Malvern). The viscosity of PBS1x is 0.87 cP and the refractive index is 1.33. The refractive index of the particles was taken to be 1.52. Data were recorded as an average of 13 five-second acquisitions. Measurements were performed in triplicate at 25 °C. Recorded data were analysed in number with the Zetasizer software, which also calculated the polydispersity index of the samples (ranging from 0 for a perfectly monodisperse homogeneous sample to 1 for a highly polydisperse heterogeneous sample).

### Transmission electron microscopy (TEM) ultrastructural analysis

*Lactococcus lactis* bacteria were transferred to aluminium sample holders and cryoimmobilized immediately using a Leica High-Pressure Machine (HPM 100, Leica Microsystems, Vienna, Austria), and then transferred to liquid nitrogen. Samples were then freeze substituted in a Leica AFS system (Leica Microsystems, Vienna, Austria) with 1% OsO4 in anhydrous acetone with 1% glutaraldehyde, and 1% water at – 90 °C for 1 day, followed by slow warming to room temperature over a period of 7 days. After rinsing in several acetone washes, samples were then gradually infiltrated with mixtures of acetone/epoxy resin and pure epoxy resin (EMbed 812 resin kit, Electron Microscopy Sciences, Hatfield, United States) for 31 h. Samples were embedded in fresh Epon and polymerized at 60 °C for 48 h. Ultrathin Sects. (90 nm) were cut on a Reichert Ultracut E ultramicrotome (Leica, Rueil-Malmaison, France), examined in transmission electron microscope (HITACHI H7800, Japan) operating at 80 kV, and photographed with an AMT nanosprint 43 camera (AMT, Woburn, USA) on the DIMACELL platform (INRAE, Dijon, France).

### Immuno-electron microscopy (IEM)

For immunolabeling of high-pressure frozen samples, the freeze substitution medium consisted of anhydrous acetone containing 0.2% uranyl acetate in the AFS unit as described above at − 90 °C for 4 days, followed by slow warming to − 50 °C over a period of 2 days. After rinsing in several acetone washes, samples were infiltrated in Lowicryl^®^ HM20 resin (MonoStep HM20 resin, Electron Microscopy Sciences, Hatfield, United States) at − 50 °C, polymerized under UV light, and subsequently sectioned. Ultrathin sections (82 nm) were cut as above and were collected onto carbon-collodion-coated 200-mesh grids. A solution of caveolin antibody diluted at 1/75 and of a goat anti-mouse conjugated with 5-nm colloidal gold diluted 1/25 (secondary antibody) were successively applied prior to TEM observations. These observations were carried out using an electron microscope (HITACHI H7800, Japan) as described above.

### Negative staining electron microscopy

Five μL of membrane fraction sample was placed on an effluved carbon formvar grid and allowed to rest for 20 min before blotting with filter paper. Samples were negative stained with commercial solution (Uranyless EMS, USA) during 3 min before blotting and air drying. Transmission electron microscopy (TEM) images were taken with a Hitachi H7800 at an acceleration voltage of 100 kV and an AMT camera.

## Results

### Cloning of Caveolin-1β within the pNZ8148 vector

In the present study, we chose to express the caveolin-1β isoform X1 from *Canis lupus familiaris*. This isoform displays 93.5% of identity with the human gene. The 10His-tag affinity tag has been added to the N-terminus of the gene to help further detection and affinity purification of the protein, and due to the involvement of the C-terminus for the conformation and functionality of the protein [34].

To generate recombinant *L. lactis* strains, cDNA encoding for the protein of interest was subcloned into pNZ8148 vector. This vector possesses the nisin inducible promoter with the obligatory *Nco*I site for translational fusions [35]. Since the pKL/HTC recombinant vector does not hold the *Nco*I site at the ATG of the gene, a mutagenesis was first performed in *E. coli* to insert this restriction site simultaneously with a codon encoding for glycine between the ATG and the second codon of the gene. After digestion with restriction enzymes and ligation, the clone pNZ8148-HTC was successfully generated (called pNZ-HTC).

### Caveolin-1β is expressed at high levels in *L. lactis* membranes

**Fig. 1:**
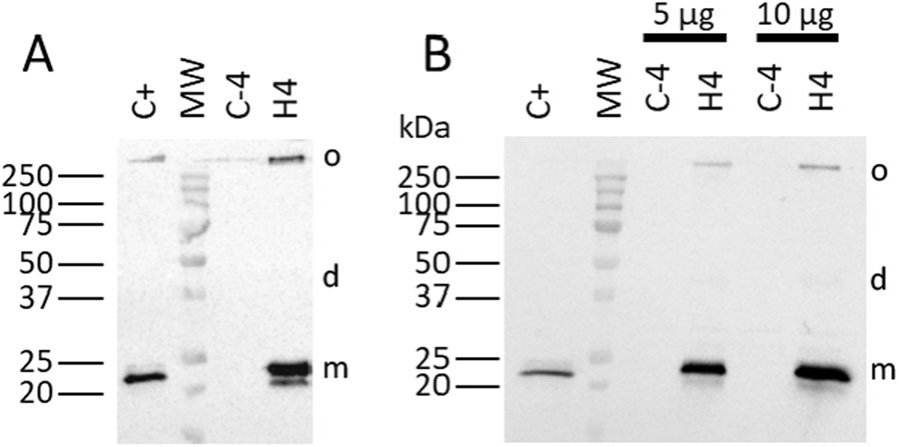
Expression of HTC in *L. lactis* after 4 h post-induction by nisin. Total membrane proteins (5 µg for **A**, 5 and 10 µg for **B**) were separated in a 12% SDS–PAGE and analyzed by Western blot performed using either an antibody specific to caveolin-1 (**A**; 1/7500) or an HRP-conjugate specific to the His-tag affinity tag (**B**; 1/5000). A positive control protein containing caveolin-1β (C + ; 15 µg) and the band of 75 kDa of the molecular weight from Bio-Rad (constitutively His-tagged) were used to estimate the expression levels of the recombinant proteins. H means membrane proteins derived from bacteria containing the recombinant pNZ-HTC vector, C- means crude membrane proteins derived from control bacteria containing the empty pNZ8148 vector. o,d,m correspond respectively to oligomer, dimer and monomer. Western blot images are merged images of both colorimetric analysis of membranes revealing the molecular weights and chemiluminescent analysis revealing only some molecular weight bands

After cloning into pNZ8148, the expression of caveolin-1β has been tested in *L. lactis*. After 4 h of nisin induction, bacteria were disrupted, and both soluble and membrane proteins were analyzed by Western blot with antibodies specific either to the protein (Fig. 1A) or to the affinity tag used (Fig. 1B).

Caveolin-1β was successfully expressed in *L. lactis* and was only present in the membrane fractions. Indeed, Western blot analysis of soluble fractions using the same antibody did not show any band (data not shown). No band was observed in the negative control (C-4) corresponding to the proteins isolated from bacteria transformed by the empty vector. We noticed the presence of additional bands at around 45 and 250 kDa, at the interface between the stacking and concentration parts of the SDS–PAGE, corresponding to the dimer and oligomers of caveolin-1β respectively. Absence of heating of the samples before the SDS–PAGE led to an increase of the amount of these oligomers from about 10% to about 50% of the total caveolin-1β expression (Additional file 1: Fig. S1). Following two independent protein quantification in triplicate using either the intensity of the 75 kDa band from Bio-Rad molecular weight (161–0373) or the positive control from Sf21 cells, the recombinant protein produced has been consistently estimated to represent about 25% of total MPs (Fig. 1A and B), a remarkably high expression level for an eukaryotic MP expressed in *L. lactis*.

Subsequently, different induction times, including shorter and longer times, were tested to check the impact of induction time on protein expression level.

**Fig. 2:**
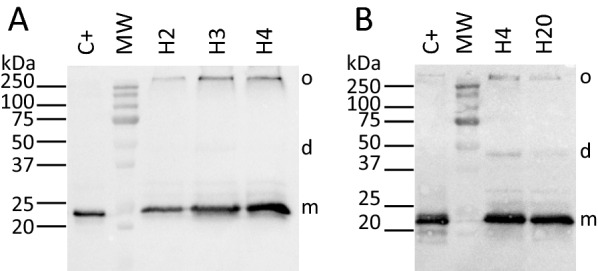
Expression of HTC in L. lactis depending as a function of induction time. **A** Impact of short times of induction on protein expression (2-, 3- and 4-h post-induction). **B** Impact of long time of induction on protein expression (4- and 20-h post-induction). Total membrane proteins (10 µg) were separated in a 12% SDS–PAGE and analyzed by Western blot performed using an HRP conjugate specific to the His-tag affinity tag. A positive control protein containing caveolin-1β (C + ; 15 µg) was loaded and the band at 75 kDa of the molecular weight from Biorad was used to estimate the expression levels of the recombinant protein. o,d,m correspond respectively to oligomer, dimer and monomer

Interestingly and as expected, the amount of protein produced was higher after 4 h of induction, as depicted on Fig. 2A. This duration has already proved to be optimal for the expression in *L. lactis* of various MPs of diverse origins [27, 33]. This time of duration was thus chosen for all the further experiments performed within this study, except for microscopy. The amount of protein expressed after 20 h post-induction was also studied to verify the amount of protein produced prior to microscopic analysis. As depicted on Fig. 2B, this amount was still relatively high, almost similar to those obtained after 4 h of induction, and appropriate for further microscopic analysis on bacteria. Moreover, additional bands corresponding to dimer and oligomer were also observed.

In conclusion, the protein caveolin-1β can be heterologenously expressed in *L. lactis*, at high levels and under its oligomeric state.

### Caveolin-1β is found in membranes of different densities after discontinuous gradient

**Fig. 3:**
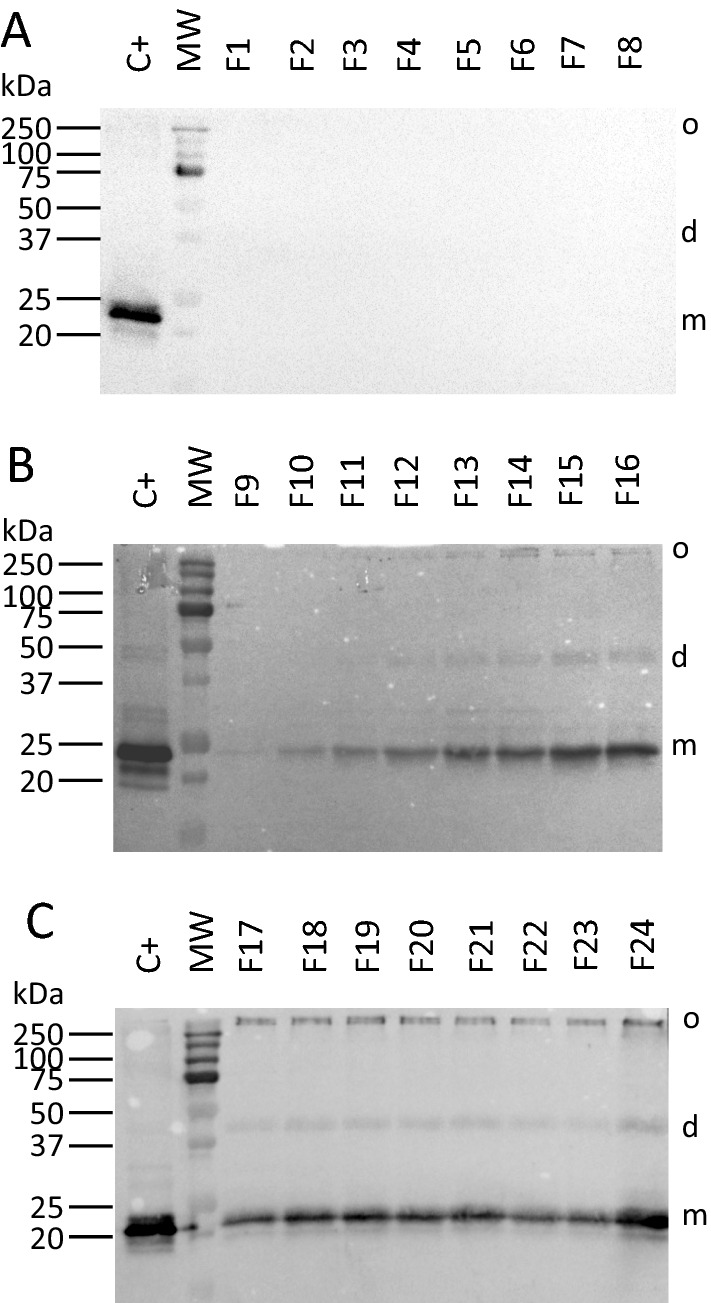
Expression of HTC in fractions after sucrose gradient. **A** Expression in fractions from 1 to 8. **B** Analysis of fractions from 9 to 16. **C** Fractions from 17 to 24. Equal volumes of fractions (20 µL) were separated in a 12% SDS–PAGE and analyzed by Western blot performed using an HRP-conjugate specific to the His-tag affinity tag. A positive control protein sample containing caveolin-1β (C + ; 15 µg) was loaded. o,d,m correspond respectively to oligomer, dimer and monomer

Equal quantities of total MPs (1.25 mg) from 3 different experiments were loaded on discontinuous sucrose gradient to separate the membranes collected after cell disruption depending on their density. In Fig. 3 are presented the Western blotting results of the different fractions obtained from one experiment representative of three.

A band at around 20–25 kDa, like that found in the positive control, was found in the fractions from 10 to 24, but absent in the first lighter fractions (from 1 to 8, a weak band in 9). The bands corresponding to the dimer (45 kDa) and the oligomer (250 kDa) started to appear in fraction 12 and in fraction 14 respectively and remained present in all the denser fractions. The intensity of all bands was higher in the final fraction 24 that corresponded to the pellet of the gradient. No band was observed in the fractions isolated from the negative control.

The presence, in fractions of different densities, of caveolin-1β in its oligomeric state suggested that the protein could possibly be functional, according to the literature reporting on the necessary caveolin-1 oligomerization for inducing membrane curvature [8, 9] (see Discussion section).

### *Lactococcus lactis* produces caveolin-1β enriched intracellular vesicles

Both caveolin-1β expressing and control bacteria were analyzed through transmission electron microscopy (TEM) 20 h post-induction to see whether the bacteria were able to produce intracellular vesicles and their subcellular localization. Immunolabelling and cryo-TEM were used to observe the presence of such vesicles and to determine their diameter.

**Fig. 4:**
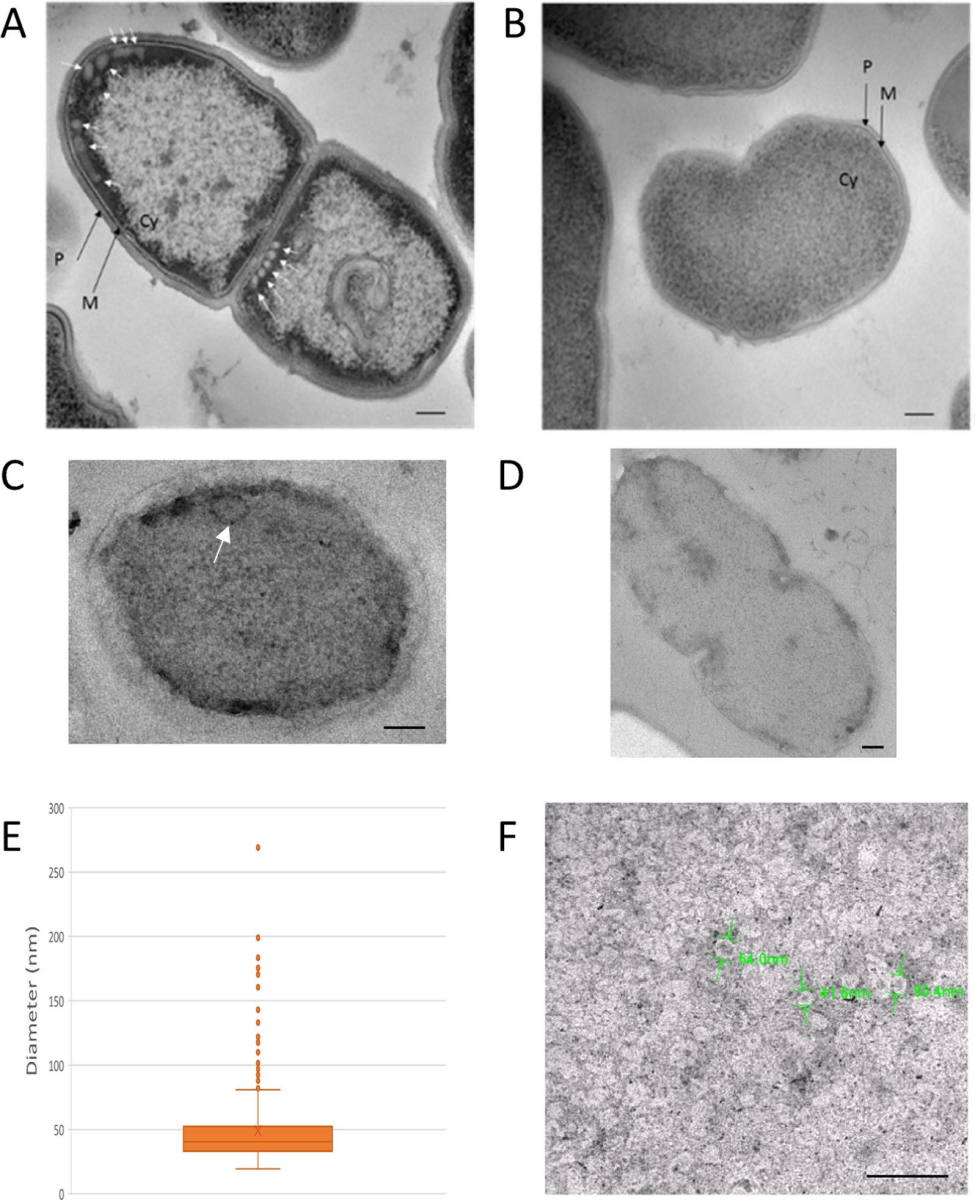
Transmission electron microscopy (TEM) analysis. **A** and **B** Cryo-TEM of recombinant bacteria containing either pNZ-HTC or the empty vector (C-). A. TEM of HTC bacteria displayed the presence of vesicle near the plasma membrane. **B** TEM of C- bacteria. Scale = 100 nm (**A** and **B**). **C** and **D** Immunolabelling of bacteria harboring either HTC (**C**) or the empty vector (C-; **D**). Scale = 100 nm. E. Distribution of diameters of intracellular caveolin-1β enriched vesicles determined from TEM images (n = 390; N = 2); the orange region depicts the 75% most frequent values. **F** Negative staining-TEM images from vesicles isolated through density discontinuous gradient fraction F15 (scale = 200 nm). P = peptidoglycan, M = plasma membrane, Cy = cytoplasm

Remarkably, vesicles were present close to the plasma membrane, mostly at the apex of HTC bacteria (Fig. 4A), whereas none was observed in the negative control bacteria (Fig. 4B). Immunolabelling was performed, using the same antibody as that used for Western blot analysis, and revealed the localization of these antibodies within the intracellular vesicles (Fig. 4C), while no immunodetection was observed in the plasma membrane devoid of such internal vesicles (Fig. 4D). All the observed vesicles (n = 390) were measured, allowing to determine a mean diameter of 50.3 ± 18.7 nm (Fig. 4E), and to estimate that almost 75% of vesicles displayed a diameter between 30 to 60 nm, with a median value of 40.2 nm. The number of vesicles contained in all the entirely imaged bacteria was determined to be 351 in 77 whole and entire bacteria, corresponding to a mean number of 4.6 vesicles/cell. However, TEM imaging can only detect vesicles present within the observed slice of 90 nm thickness, cut from a whole bacterium of diameter roughly ten times larger, and this requires a geometric correction for evaluating the number of intracellular vesicles (Additional file 1: Fig. S2). Assuming in a first approach a spheric bacteria and an equatorial cylinder of the same diameter for modeling the TEM section, the volume ratio is about 7, meaning that we must consider that there are about 30 vesicles in each *L. lactis* cell overexpressing caveolin-1β. For the sake of comparison with the vesicles isolated from these bacteria after their disruption, negative staining-TEM analysis of the fraction F15, corresponding to 31.25% sucrose, displayed vesicles with diameters of 52 ± 8 nm (n = 102) (Fig. 4F).

### Vesicles containing caveolin-1β display different diameters after discontinuous density gradient

**Fig. 5:**
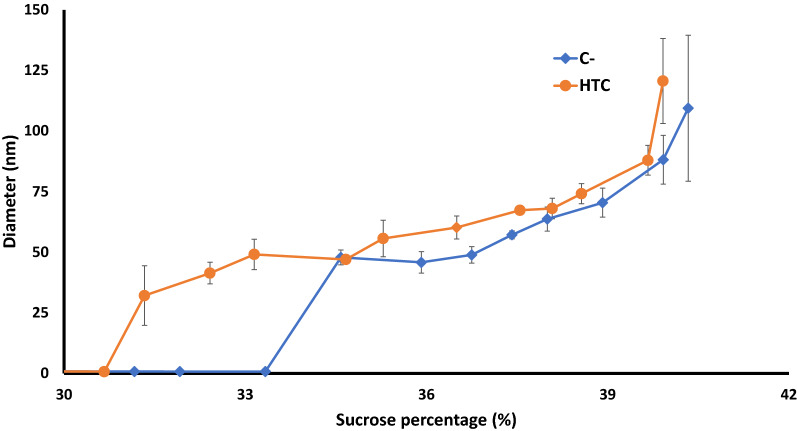
DLS analysis of fractions depending on sucrose percentage in fractions. Fractions were diluted 1/100 in PBS1× pH7.4 prior to analysis. HTC (orange circles) and C- (blue diamonds). N = 3; results are mean of 9 different measurements since the NanoZS apparatus perform three determinations per sample

After biochemical analysis, the fractions were analyzed by dynamic light scattering (DLS) to determine the vesicle diameter. The fractions from both types of total MPs (C- and HTC) of three independent experiments were analyzed (Fig. 5).

HTC and C- fractions displayed different size profiles. First, no vesicle was detected and measured in fractions F1-F13 up to 31% of sucrose for both types of fractions, although some amounts of MPs were readily present when assayed in these fractions (data not shown). Then, for the fractions F14-F16 between 31 and 34% sucrose, vesicles from 30 to 50 nm (mean diameter 40.9 ± 8.0 nm) were detected and measured only in the HTC fractions, but not in the C- fractions. Their polydispersity indexes were measured between 0.25 and 0.30, revealing a relatively high homogeneity in size. Then, for increasing densities above 34.5% sucrose, fractions F17-F24 of both HTC and C- bacteria displayed vesicles with increasing diameters, from 50 to about 120 nm. At each density, vesicles from HTC fractions displayed a tendency for a higher diameter than those from C- fractions (almost 7 to 10 nm of difference).

## Discussion

By comparison with the few eukaryotic and prokaryotic expression systems already tested and reported [16–18, 21], the heterologous expression of caveolin-1β in the Gram^+^ bacterial host *L. lactis* has allowed to draw some clues about its relationships within its membrane environment.

### Caveolin-1β is expressed at a remarkably high level for an eukaryotic MP expressed in *L. lactis*

Caveolin-1β expression was tested in *L. lactis* bacteria using the NICE system. After 4 h of nisin induction, bacteria were able to express caveolin-1β at an unexpectedly high expression level for an eukaryotic MP, around 25% of total MPs (Fig. 1A, B), when compared to other eukaryotic MPs previously expressed in the same bacterial host, i.e. typically between 1 to 5% of total MPs [30]. Comparison of this relatively high production yield with that obtained for different forms of caveolin within other heterologous expression systems is difficult since quantified data are not available [16–18, 21].

The relatively high expression of caveolin-1β we obtained could be linked to different features of the protein and/or the host *L. lactis*.

First, it could be related to the caveolin-1β conformation and insertion characteristics within the lipid bilayer. As a matter of fact, only one eukaryotic MP has been expressed in *L. lactis* at a high level comparable to that obtained with caveolin-1β, the plant chloroplastic oxoene reductase ceQORH [33]. This extrinsic protein associates with *L. lactis* plasma membrane by interacting with the membrane polar interface through similar electrostatic interactions as in its native organelle, chloroplast [36]. Other proteins belonging to the same family have been shown to be also dimeric, possibly also the case for the ceQORH in a favorable environment such as *L. lactis* even if it was monomeric in *E. coli* [37]. By analogy, it could be considered that caveolin-1β, which is a small MP inserted only into one leaflet of the membrane by a helix-break-helix motif, would rather easily find a “well-suited” membrane environment in *L. lactis*. Anyway, thanks to its high level of production in a functional, membrane curving state, our data open the way to the determination of caveolin-1β structure using *L. lactis* as a bacterial host.

Second, this relatively high expression level could also be linked to the specific lipid composition of *L. lactis*. Prakash et al. [38] highlighted the importance of cholesterol, phosphatidylserine [21, 39], sphingomyelin and glycosphingolipids in caveolin-1 mediated membrane curvature. Yet *L. lactis* does not possess such lipids within its membrane, but in contrast it displays notably large amounts of cardiolipin (42.5% mol/mol) [40]. This lipid has already been demonstrated to play an important role in MP stability [41], microdomain formation [42] and membrane curvature [43], some properties shared with cholesterol [10]. Hence, cardiolipin could act to stabilize caveolin-1β within the membrane, therefore favoring its expression and functionality to induce intracellular vesicles formation within bacteria. However, it should be noted that caveolin-1β is believed to insert into the external leaflet of the vesicles, due to a wedge effect [44], whereas cardiolipin is expected to segregate within its internal leaflet of the membrane (due to its small polar headgroup). This means that such stabilizing effect of cardiolipin on caveolin-1β should be considered as indirect, even if potentially relevant for helping it to contribute for generating membrane curvature. Indeed, the intracellular caveolar vesicles were observed by EM to be mainly present at the apex of the cells, in particular at the bacteria division sites (Fig. 4A), a region where the plasma membrane is rather curved and reported in *E. coli* to be enriched in cardiolipin [42, 45, 46]. The fact that these vesicles were observed at the septum of dividing bacteria is reminiscent of a similar localization for eukaryotic caveolae in non-transfected organisms, where caveolin and caveolae are involved in membrane changes during abscission and cytokinesis [47]. Finally, this situation of MP-lipid relationships is possibly an illustration of the important role of cardiolipin that has been involved for intracellular curved membrane structures formation in *E. coli* when overexpressing some specific MPs [48, 49]. Further lipidomic analyses of light and dense vesicles could help to characterize them and to understand the formation of caveolin-1β enriched intracellular vesicles in *L. lactis*.

### Virtually all expressed oligomerized caveolin-1β is harbored by intracellular neo-formed vesicles

Electron microscopy (EM) imaging has revealed that the HTC-expressing bacteria presented several intracellular vesicles presenting fairly homogenous size centered around 40–50 nm of diameter (Fig. 4A), while this was not observed in the control cells (Fig. 4B). This illustrates the capacity of caveolin-1β to induce membrane remodeling (directly or not). The location of these vesicles beneath the plasma membrane indicates that they are likely formed from it, or at least from certain sub-domains of the bacterial plasma membrane since they appeared to be not equally distributed along all the cell membrane. The immuno-labelling experiments further pointed to a clear location of caveolin-1β at the level of these intracellular vesicles, but no labelling of the plasma membrane at distant sites of these vesicles could be detected (Fig. 4C). This shows that caveolin-1β is sufficient, in the absence of any of the protein partners found in mammalian cells exhibiting caveolae, such as cavins, and of bacterial homologues, for being directly responsible for a local membrane curving effect leading to vesiculation with a high efficiency. In addition, EM imaging allowed to grossly evaluate the total quantity of membranes corresponding to the cumulated number of these intracellular vesicles within a transformed bacterium, taking roughly 30 vesicles/cell. Indeed, considering this number and their mean diameter leads to a cumulated area of about 240,000 nm^2^ for the total membranes of these intracellular vesicles in each bacterium. The imaged bacteria harbored a total plasma membrane area of about 2 µm^2^ for the round “interphasic” ones (c.a. 0.8 µm diameter), while the much more elongated “pre-division” ones (c.a. 0.6 µm width and 1.3 µm length) had an area of about 4 µm^2^. These values lead to evaluate that the total amount of membranes of these intracellular vesicles represent about 6 to 12% of the plasma membrane of the transformed HTC bacteria. These neo-formed intracellular vesicles can thus well accommodate the overexpressed oligomerized caveolin-1β, evaluated to be roughly almost the half of the total expressed caveolin-1β and thus about 12–13% (for a total of about 25%). This is thus consistent with the fact that a large majority of the expressed oligomerized caveolin-1β is present in these internal vesicles, which then could be so-called “caveolar vesicles”. We propose this term as a proper description for these membrane structures and consider that it is better suited than the previously used appellation “heterologous caveolae” since there is no morphological indication of caveolin-1β expression at the level of the plasma membrane. They are similar in size to the intracellular vesicles produced in *E. coli*, i.e. 45–50 nm diameter [17], but smaller than those produced in insect cells [18], in *Toxoplasma gondii* [21], as well as in the native caveolae [50] that were reported to be 60 to 90 nm of diameter.

### Caveolar vesicle formation is correlated with caveolin-1β oligomerization

*Lactococcus lactis* was able to express caveolin-1β in its oligomeric state, since both dimeric and oligomeric forms were observed on the Western blot analyses of both HTC total MPs and membrane fractions (Figs. 1–3). Indeed, *L. lactis* can express various MPs under their native oligomeric state [25, 27]. The production of such forms underlies the possibility of expression of a functional caveolin-1 able to induce the formation of intracellular vesicles. Only few studies on caveolin-1 expression displayed data on the various forms of the protein and their analyses through Western blots [23, 51]. The oligomers are larger than 250 kDa and found at the interface between the two types of SDS–PAGE gels (concentration and separation), even if the samples were heated at 95 °C for 10 min in 4% SDS. This phenomenon could be explained by the fact that oligomers are highly resistant to detergents as pointed by Zhang et al. [23] which suggested to boil them prior to SDS–PAGE. Supplementary experiments shown that boiling samples led to a large denaturation of the oligomers (Additional file 1: Fig. S1). The fact that some oligomers were still present after boiling could be due to the relatively high amount of caveolin-1β produced in *L. lactis*.

Interestingly, we observed thanks to membrane fractionation that the caveolin-1β oligomers could be detected from the fraction F14 and in all the denser ones (Fig. 3), which is in perfect correlation with the appearance of membrane vesicles from HTC-expressing bacteria as detected by DLS (Fig. 5). This observation thus provides strong indication for the requirement of caveolin-1β oligomerization for the intracellular caveolar vesicles formation. By inference, caveolin-1β monomers (and dimers) should be present in limited amounts in the plasma membrane, where they hence can be hardly immuno-detected.

### The formed caveolar vesicles display a certain heterogeneity in density

Data obtained by Western blotting and DLS analysis of membrane fractions have been combined to analyze the nature of vesicles isolated and to distinguish the different types of populations observed by DLS analysis. For the control bacterial cells, DLS measurements have shown that the lighter membrane fractions F1 to F16 did not display any detectable vesicles, although they contain nearly the half of the MPs (data not shown). This shows that a notable part of the disrupted plasma membrane produced non-vesiculated membrane fragments of low density, and only the denser fractions (F17 to F24) were able to form revesiculated membranes after the cell disruption. In the membrane fractions obtained from HTC-expressing bacteria, the light ones F10–F13 contained caveolin-1β monomers (and some dimers) but no vesicle structures, while DLS could detect membrane vesicles in the fractions F14–F16. This means that these light vesicles, containing caveolin-1β (with some oligomers, as stated above) are formed before bacterial cell disruption, and are attributable to the lightest part of the intracellular caveolar vesicles. In the denser fractions F16 to F24, there is thus a coexistence of denser caveolar vesicles and revesiculated plasma membrane fragments.

Thus, density sucrose fractionation allowed separating two types of vesicles containing caveolin-1β out of the total MPs, i.e. small and light vesicles of 30 to 50 nm of diameter and larger vesicles from 50 to 120 nm in denser fractions. The light caveolar vesicle fraction F15 has been observed by EM, and this allowed to measure their mean size, which was found in good agreement with the DLS data. These light caveolar vesicles being most probably characterized by a lower protein-to-lipid ratio than the denser ones, this means they should contain only few endogenous MPs, if any, (which otherwise cannot revesiculate after cell disruption) besides caveolin-1β oligomers, considering that caveolin oligomers are reported to exert their membrane curving effect according to rather defined stoichiometric ratios [1, 8, 45]. In contrast, the denser caveolar vesicles, hence with a higher protein-to-lipid ratio and presenting a somehow constant number of oligomers immuno-detected in fractions F17 to F24 (Fig. 3C), should contain some higher amounts of endogenous MPs. This progressive change in MP composition, from F17 to F24, happened to be correlated with a significant increase of the vesicle size as measured by DLS (Fig. 5), even if this technique cannot per se separate the respective contributions of the two vesicle populations coexisting in these density fractions. Of note, our study is one of the rare one determining caveolar vesicle diameter through DLS, since most reports of vesicle diameter determinations were performed through TEM analyses. This technique allows diameter measurements in solution, less perturbing for biological samples including vesicles. The present study followed the “MISEV guidelines” for vesicle analysis since two complementary techniques (DLS and cryo-TEM) have been used for the analysis and gave comparable results [52]. Moreover, polydispersity indexes of vesicles obtained through DLS analysis were relatively low (around 0.3), while working with biological samples, which highlights the “high quality” in term of size of vesicles produced by *L. lactis*. However, DLS only allows measurement of mean vesicle diameters but does not provide information on the concentration of these vesicles in each fraction, especially in case of size heterogeneity. If available, this additional information would provide insights on the mode of production of these vesicles within the bacteria, and in particular allowing to estimate the proportion of light and dense caveolar vesicles without the need to biochemically separate them. Currently, almost none of the techniques of determining vesicle concentration can reliably measure vesicles smaller than 50 nm in diameter in solution [53]. Future analyses by different techniques, alone or in combination, are needed to refine these aspects.

The production by *L. lactis* of internal dense caveolar vesicles containing various endogenous MP normally resident in the plasma membrane (preliminary proteomic data not shown) provides the indication that caveolin-1β biosynthesis occurs, at the level of the plasma membrane, in domains that are shared with the biosynthesis of at least some of the MPs of the bacterial host. Such functional domains appear thus similar to the so-called transertion domains described for *E. coli* and *Bacillus subtilis*, that allow concerted transcription, translation and membrane insertion of neo-synthesized MPs [54]. These membrane domains have been recognized to play a pivotal role for the appearance of various ectopic intracellular membrane structures induced by overexpression of some MPs in bacteria [49]. Indeed, these domains allow the recruitment in the close membrane vicinity of the neo-synthesized caveolin-1β of various MPs before vesiculation occurs. Consequently, this will lead to the formation of a new intracellular membrane compartment harboring some endogenous plasma membrane MPs, the nature of which depending on their affinity with the locally selected lipids and on their ability to accommodate within a curved membrane. In a prospective frame, this opens the interesting possibility that heterologous co-expression of caveolin-1β with another MP (whatever its biological origin) could lead this MP to be similarly handled within these neo-formed intracellular caveolar vesicles.

## Conclusion

Interestingly, *L. lactis* has been shown not only to express caveolin-1β at an unexpectedly high expression level for an eukaryotic MP, but also under its functional oligomeric form, allowing the formation of intracellular vesicles from 30 to 60 nm. Since *L. lactis* does not possess the protein partners and the lipids known to be necessary for caveolae formation, further investigations involving proteomic and lipidomic analyses would allow to decipher the molecular mechanisms involved in the formation of these neo-formed intracellular vesicles in *L. lactis*. Moreover, such heterologous nanovesicles could provide biological materials well-suited for improving MPs functional and structural characterization, as well as they could be used for various biotechnological purposes, including delivery of therapeutics.

## Supplementary Information

**Additional file 1:** **Figure S1.** Expression of HTC in *L. lactis* after 4 hours post-induction by nisin. Total membrane proteins (5 µg for panel A, 5 and 10 µg for panel B) were separated in a 12% SDS–PAGE and analyzed by Western blot performed using either an antibody specific to caveolin-1 (A; 1/7500) or an HRP-conjugate specific to the His-tag affinity tag (B; 1/5000). A positive control protein containing caveolin-1β (C+) and the band of 75 kDa of the molecular weight from Bio-Rad (constitutively His-tagged) were used to estimate the expression levels of the recombinant proteins. H means membrane proteins derived from bacteria containing the recombinant pNZ-HTC vector, C- means crude membrane proteins derived from control bacteria containing the empty pNZ8148 vector, * means samples loaded without boiling step. o,d,m correspond respectively to oligomer, dimer and monomer. Western blot images are merged images of both colorimetric analysis of membranes revealing the molecular weights and chemiluminescent analysis revealing only some molecular weight bands. **Figure S2.** The red spots depict the intracellular caveolar vesicles, whose number is to be evaluated from their count observed by electron microscopy using a thin slice of the cell. The *L. lactis* cell (blue) is considered as a sphere of diameter 2r, and the slice cut for electron microscopy observation (black and grey) is considered as an equatorial cylinder of diameter 2r and thickness h (h = 90 nm). Cell volume is : V = (4/3) π r^3^ ; cylinder volume is : v = π r^2^ h. The volume ratio is : V/v = 4r/3h. Assuming 2r ≈ 10 h, V/v ≈ 7. In the case of the section is not in the equatorial plane of the cell, the ratio V/v is even slightly larger.

## Data Availability

The datasets generated during and/or analysed during the current study are available from the corresponding author on reasonable request.
