# Supplementary material for: Formation of intracellular vesicles within the Gram+ Lactococcus lactis induced by the overexpression of Caveolin-1β
Source: Microb Cell Fact. 2022 Nov 17;21:239. doi: 10.1186/s12934-022-01944-9 (PMC9670397; doi:10.1186/s12934-022-01944-9)
Supplement: Supplementary file 1 — Additional file 1: Figure S1. Expression of HTC in L. lactis after 4 hours post-induction by nisin. Total membrane proteins (5 µg for panel A, 5 and 10 µg for panel B) were separated in a 12% SDS–PAGE and analyzed by Western blot performed using either an antibody specific to caveolin-1 (A; 1/7500) or an HRP-conjugate specific to the His-tag affinity tag (B; 1/5000). A positive control protein containing caveolin-1β (C+) and the band of 75 kDa of the molecular weight from Bio-Rad (constitutively His-tagged) were used to estimate the expression levels of the recombinant proteins. H means membrane proteins derived from bacteria containing the recombinant pNZ-HTC vector, C- means crude membrane proteins derived from control bacteria containing the empty pNZ8148 vector, * means samples loaded without boiling step. o,d,m correspond respectively to oligomer, dimer and monomer. Western blot images are merged images of both colorimetric analysis of membranes revealing the molecular weights and chemiluminescent analysis revealing only some molecular weight bands. Figure S2. The red spots depict the intracellular caveolar vesicles, whose number is to be evaluated from their count observed by electron microscopy using a thin slice of the cell. The L. lactis cell (blue) is considered as a sphere of diameter 2r, and the slice cut for electron microscopy observation (black and grey) is considered as an equatorial cylinder of diameter 2r and thickness h (h = 90 nm). Cell volume is : V = (4/3) π r3 ; cylinder volume is : v = π r2 h. The volume ratio is : V/v = 4r/3h. Assuming 2r ≈ 10 h, V/v ≈ 7. In the case of the section is not in the equatorial plane of the cell, the ratio V/v is even slightly larger. [file 12934_2022_1944_MOESM1_ESM.docx]

**Figure S1:** Expression of HTC in *L. lactis* after 4 hours post-induction by nisin. Total membrane proteins (5 µg for panel A, 5 and 10 µg for panel B) were separated in a 12% SDS-PAGE and analyzed by Western blot performed using either an antibody specific to caveolin-1 (A; 1/7500) or an HRP-conjugate specific to the His-tag affinity tag (B; 1/5000). A positive control protein containing caveolin-1β (C+) and the band of 75 kDa of the molecular weight from Bio-Rad (constitutively His-tagged) were used to estimate the expression levels of the recombinant proteins. H means membrane proteins derived from bacteria containing the recombinant pNZ-HTC vector, C- means crude membrane proteins derived from control bacteria containing the empty pNZ8148 vector, * means samples loaded without boiling step. o,d,m correspond respectively to oligomer, dimer and monomer. Western blot images are merged images of both colorimetric analysis of membranes revealing the molecular weights and chemiluminescent analysis revealing only some molecular weight bands.

**Figure S2**

The red spots depict the intracellular caveolar vesicles, whose number is to be evaluated from their count observed by electron microscopy using a thin slice of the cell.

The L. lactis cell (blue) is considered as a sphere of diameter 2r, and the slice cut for electron microscopy observation (black and grey) is considered as an equatorial cylinder of diameter 2r and thickness h (h = 90 nm).

Cell volume is : V = (4/3) π r^3^ ; cylinder volume is : v = π r^2^ h.

The volume ratio is : V/v = 4r/3h.

Assuming 2r ≈ 10 h, V/v ≈ 7.

In the case of the section is not in the equatorial plane of the cell, the ratio V/v is even slightly larger.
